# Nutritional status of under six years old children in Kalar city, Kurdistan Region, Iraq

**DOI:** 10.1186/s12889-022-14071-2

**Published:** 2022-09-02

**Authors:** Hawal Lateef Fateh, Mostafa Nachvak, Hadi Abdollahzad, Shahab Rezaeian, Mina Darand, Amir Bagheri

**Affiliations:** 1 Student Research Committee, Kermanshah University of Medical Sciences, Kermanshah, Iran; 2 Nursing Department, Sulaimani Polytechnic University, Kurdistan region, Sulaimani, 460001 Iraq; 3 Department of Nutrition, Research Center for Environmental Determinants of Health (RCEDH), Kermanshah University of Medical Sciences, Kermanshah, Iran; 4 Infectious Diseases Research Center, Kermanshah University of Medical Sciences, Kermanshah, Iran; 5 Student Research Committee, Isfahan University of Medical Sciences, Isfahan, Iran; 6 Student Research Committee, Tehran University of Medical Sciences, Tehran, Iran

**Keywords:** Nutritional status, Kurd children, Malnutrition, Wasting, Stunting, Iraq

## Abstract

**Introduction:**

Nutritional problems in children cause major morbidity and mortality in the world. This study aimed to assess the nutritional status of under six years old children in Kalar city, Kurdistan Region, Iraq.

**Methods:**

In this longitudinal study, data from 403 Iraqi Kurdish children aged 0–72 months and their mothers were extracted from Health Centre in Kalar city undertaken between 2013 and 2019. The children`s growth data were obtained at birth time, 6, 12, 24, and 72 months. Epi Info was used to classify the children of nutritional status by converting the anthropometric measurements into Z-scores. Data were analyzed using SPSS 25 software.

**Results:**

The prevalence of overweight and obesity rose from birth to age 6 years old, from 19.6% and 7.4% to 52.2% and 30.5%, respectively. At 24 month, children had the highest rates of being overweight (56.1%) and obesity (34%). At 6 month, the highest prevalence of wasting exists (9.5%). At 6 month boys and girls had the highest frequency of stunting, 17.2% and 7.2% respectively. Considering the association of all characteristic variables and growth data at birth time, only mothers with academic education had children with significantly higher BMI for age compared to illiterate mothers after adjusting for all potential confounders (β: 0.573, 95% CI: 0.105, 1.04, *P*: 0.017).

**Conclusion:**

The study suggests that some analysed factors that accounted for malnutrition in Kalar city’s children are preventable. Therefore, to reduce the burden of malnutrition, community-based education and targeted nutritional interventions are required.

## Introduction

Malnutrition in children is a major public health problem in developing countries and it has both short- and long-term health consequences [1, 2]. It has an impact on children's health and development, increases the risk of illnesses, and contributes considerably to morbidity and death in children [3, 4]. Three well-known markers of a child's nutritional condition are underweight, wasting and stunting [5]. Wasting and stunting are the indicators of under-nutrition [5, 6]. Malnutrition has a key role in the worldwide burden of several illnesses [7].

According to a global nutrition study in 2018, the prevalence of coexisting stunting, wasting, and overweight in Iraqi children under the age of five was about 4, 10.30, and 2.80 per cent, respectively [8]. Malnutrition in children is caused by a variety of risk factors, Several studies have identified socioeconomic inequalities, regional variations, poor feeding habits, family food insecurity, maternal low education, and youngster morbidity as common risk factors [9, 10].

In childhood and adolescence, natural growth is the most significant health indicator. Individually and in groups, standard growth charts and graphs are used to examine the nutritional and growth status of children and adolescents [11]. Growth trends measure the rate of change over time. They may be measured over any period, such as a month, a year, or a decade. You can anticipate future growth by identifying the growth trend [12].

The Iraqi Kurdistan region is one of the regions that has undergone extensive economic and social changes in the last three decades. The occurrence of these changes has had a great impact on children. Due to these changes, very few studies related to health and nutrition sciences have been done in this part of the world, the purpose of this study is to assess the nutritional status of children from birth time to 6 years old in Kalar city.

## Methods

### Study location and population

This longitudinal study was conducted in 2020 at the Sherwana Primary Health Centre in Kalar city, Kurdistan Region of Iraq. The participants consists of children who had born in 2013 and followed their growth during six years. A sample size of 403 children was estimated at 95% significant levels, an error level of 0.05, and 10% additional samples for considering missing data. We included healthy children who were all free of congenital abnormalities and had lived in Kalar city for a year or more. However, children due to having a congenital abnormality, discontinuing follow-up, living in rural areas or living in the study area for less than 1 year were excluded from the study.

### Data collection

Demographic data from the family certificate included the mother's age (year), family income, type of delivery, abortion history, gestational diabetes, consanguinity marriage, supplementation, anemia during pregnancy, unwanted pregnancy, twins, mother's job, father's job, and maternal education. The height (cm) and weight (kg) of the study children were measured using a measuring board (to the nearest 0.1 cm) and a standard weight scale (to the nearest 0.1 kg). When the measurements were collected, the children were dressed simply and wore no shoes. The ages of the children were obtained from their birth certificates.

Based on the WHO growth standards, nutritional disorders were defined as following [13]:

- Stunting: height for age < –2 SD of the WHO Child Growth Standards median.
- Wasting: BMI for age < –2 SD of the WHO Child Growth Standards median.
- Overweight: BMI for age > +2 SD of the WHO Child Growth Standards median.
- Obese: BMI for age > +3 SD of the WHO Child Growth Standards median.

### Statistical analysis

The samples were statistically analyzed at a 95% significant level using SPSS software version 25. Epi Info version 7 was used to classify the study children of nutritional status by converting the anthropometric measurements into Z-scores. The prevalence of this malnutrition status is calculated as the number of cases divided by the children population. One sample Z-test was used for comparing the Z-score distribution of weight for age (WAZ), height for age (HAZ) and BMI for age (BAZ) with a standard index of WHO. Univariate and multiple linear regression models were used to assess the factors associated with WAZ, HAZ and BAZ. Those variables with a *P*-value lower than 0.2 in the univariate analysis (crude analysis) were entered into the multiple models (adjusted model). The samples were statistically analyzed at a 95% significant level using SPSS software version 25.

## Results

The characteristics of the participants are shown in Table 1. A total of 403 mothers and their children from a total of 463 subjects were included in the study. 84.12% of mothers were housewives, and 20.35% of them had low income status. In terms of gender, 58.1% of children were female.

**Table 1:** Characteristics of study subjects and associated factors with height for age and BMI for age at birth time

			Height-for-age			BMI-for-age		
		N (%)	Crude analysis			Crude analysis		
Variable^*^	Subgroup		Coefficient	95% CI	*P*-value	Coefficient	95% CI	*P*-value
Mother’s age (year)	< 30	221 (54.8)	Ref	-	-	Ref		
	> 30	182 (45.2)	0.18	-0.04,0.42	0.120	-0.09	-0.40, 0.21	0.560
Family income	High	69 (17.12)	Ref	-	-	Ref		
	Normal	252 (62.53)	0.04	-0.27,0.36	0.774	0.37	-0.04, 0.78	0.080
	Low	82 (20.35)	0.30	-0.07,0.69	0.114	0.02	-0.47, 0.52	0.926
Type delivery	Normal	291 (72.21)	Ref	-	-	Ref		
	C-Section	112 (27.79)	-0.12	-0.38,0.13	0.335	-0.05	-0.39, 0.28	0.756
Abortion history	Yes	33 (8.19)	Ref	-	-	Ref		
	No	370 (91.81)	-0.15	-0.57,0.27	0.488	0.16	-0.38, 0.72	0.550
Gestational diabetes	Positive	13 (3.23)	Ref	-	-	Ref		
	Negative	390 (96.77)	0.13	-0.52,0.79	0.692	-0.60	-1.47, 0.25	0.169
Consanguinity marriage	No	299 (74.19)	Ref	-	-	Ref		
	Yes	104 (25.81)	0.09	-0.17,0.36	0.481	0.21	-0.13, 0.56	0.222
Supplementation	Folic Acid	228 (56.58)	Ref	-	-	Ref		
	No thing	175 (43.42)	0.13	-0.10,0.36	0.266	-0.24	-0.55, 0.05	0.112
Anemia during pregnancy	No	376 (93.30)	Ref	-	-	Ref		
	Yes	27 (6.70)	-0.37	-0.84,0.90	0.114	-0.39	-1.00, 0.22	0.210
Unwanted pregnancy	No	37 (9.18)	Ref	-	-	Ref		
	Yes	366 (90.82)	0.26	-0.37,0.43	0.898	0.03	-0.49, 0.56	0.899
Twins	Yes	15 (3.72)	Ref	-	-	Ref	-	-
	No	388 (96.28)	-0.37	-0.99,0.24	0.231	0.23	-0.56, 1.04	0.561
Mother job	Houswife	399 (84.12)	Ref	-	-	Ref		
	Employed	64 (15.88)	0.29	-0.02,0.61	0.071	-0.23	-0.64, 0.18	0.278
Father job	Jobless	64 (15.88)	Ref	-	-	Ref		
	Manual worker	258 (64.02)	-0.11	-0.44,0.21	0.486	0.12	-0.29, 0.55	0.555
	Employed	81 (20.10)	-0.22	-0.62,0.16	0.256	-0.25	-0.77, 0.25	0.321
Mother education	No Formal	60 (14.9)	Ref	-	-	Ref		
	Primary	149 (37.0)	-0.002	-0.36,0.35	0.989	0.04	-0.41, 0.51	0.834
	Academic	194 (48.1)	-0.046	-0.39,0.30	0.791	0.63	0.18, 1.08	0.005

^*^ Data were presented as numbers (%).Data were analyzed using the regression model. The first row of each subgroup’s variables considers a reference group (Ref)

The association of all characteristic variables and Z-score of height for age and BMI for age at a birth time in the crude and adjusted analysis are depicted in Tables 1 and 2, respectively. Regarding height for age Z-score, no significant difference was observed for all demographic variables in the crude and adjusted model. Considering BMI for age Z-score, only mothers with academic had significantly higher BMI for age Z-score compared to mothers without formal education (β: 0.63, 95% CI: 0.18, 1.08, *P*-value: 0.005). This association remained significant after adjusting for all potential confounders (β: 0.573, 95% CI: 0.105, 1.04, *P*-value: 0.017). Other characteristic variables did not show a significant association.

**Table 2:** Adjusted association of characteristics variables with height for age and BMI for age at birth time

		**Height-for-age**			**BMI-for-age**		
Variable^*^	Subgroup	Coefficient	95% CI	*P*-value	Coefficient	95% CI	*P*-value
Mother’s age (year)	< 30	Ref	-	-	-	-	-
	`30	0.20	-0.04,0.46	0.108	-	-	-
Family income	High	Ref	-	-	Ref		
	Normal	-0.12	-0.49, 0.24	0.499	0.007	-0.460, 0.474	0.975
	Low	0.06	-0.37, 0.50	0.759	-0.191	-0.714, 0.332	0.474
Gestational diabetes	Yes	-	-	-	Ref	-	-
	No	-	-	-	-0.59	-1.45, 0.27	0.178
Supplementation	Folic Acid	-	-	-	Ref	-	-
	No thing	-	-	-	-0.13	-0.44, 0.17	0.387
Anemia during pregnancy	No	Ref	-	-	-	-	-
	Yes	-0.31	-0.80, 0.17	0.210	-	-	-
Mother job	Housewife	Ref	-	-	-	-	-
	Employed	0.18	-0.16, 0.54	0.295	-	-	-
Mother education	No Formal	-	-	-	Ref	-	-
	Primary	-	-	-	0.009	-0.459, 0.477	0.969
	Academic	-	-	-	0.573	0.105, 1.04	0.017

^*^ Data were analyzed using the regression model. First row of each subgroup’s variables consider as reference group (Ref.)

Height and BMI for age Z-score from birth time to 6 years old of children in comparison with WHO standard has shown in Table 3, The findings of the present study showed the highest prevalence of overweight and obesity (BMI for age) in 24 month children (62.4%) and (52.5%), respectively and *P*-value of BMI for age Z-score in boys and girls vs. WHO standard was < 0.0001. In addition, the highest prevalence of obesity and overweight was in boys. The prevalence of wasting (weight-for-height) was 4% at the birth time but it rose to 4.9% in 6 years old, *P*-value of height for age Z-score in boys and girls vs. WHO standard was < 0.0001. The lowest prevalence of stunting according to height for age was in children of 12 month (1.7%).

**Table 3:** Height for age Z-score and BMI for age Z-score from birth time to 6 years old of children in comparison with WHO standards

			**Height for age**	**BMI for age**			
		**Stunted %**	***P*-value**	**Wasted %**	**over weight%**	**Obese%**	*P-value
**At birth**	**Boy**	4.1	0.0006	1.8	17.2	4.1	< 0.0001
	**Girl**	1.23	0.0869	5.6	21.4	9.8	< 0.0001
**6 month**	**Boy**	17.2	< 0.0001	9.6	30.1	13.3	< 0.0001
	**Girl**	1.33	< 0.0001	9.5	24.6	11.6	< 0.0001
**12 month**	**Boy**	1.7	0.2872	0.6	33.1	8.3	< 0.0001
	**Girl**	0	< 0.0001	2.6	32.5	13.2	< 0.0001
**24 month**	**Boy**	5.9	0.0773	3.9	61.4	35.3	< 0.0001
	**Girl**	0.33	< 0.0001	4	52.5	33.2	< 0.0001
**72 month**	**Boy**	11.8	0.0317	3.5	59.9	38	< 0.0001
	**Girl**	7.3	0.0031	5.9	47.3	25.7	< 0.0001

Data were presented as percent (%). Data were analyzed using one sample Z-test

^*^P-Values for three indicators (wasted, over-weight and obese) were < 0.0001

**Fig. 1:**
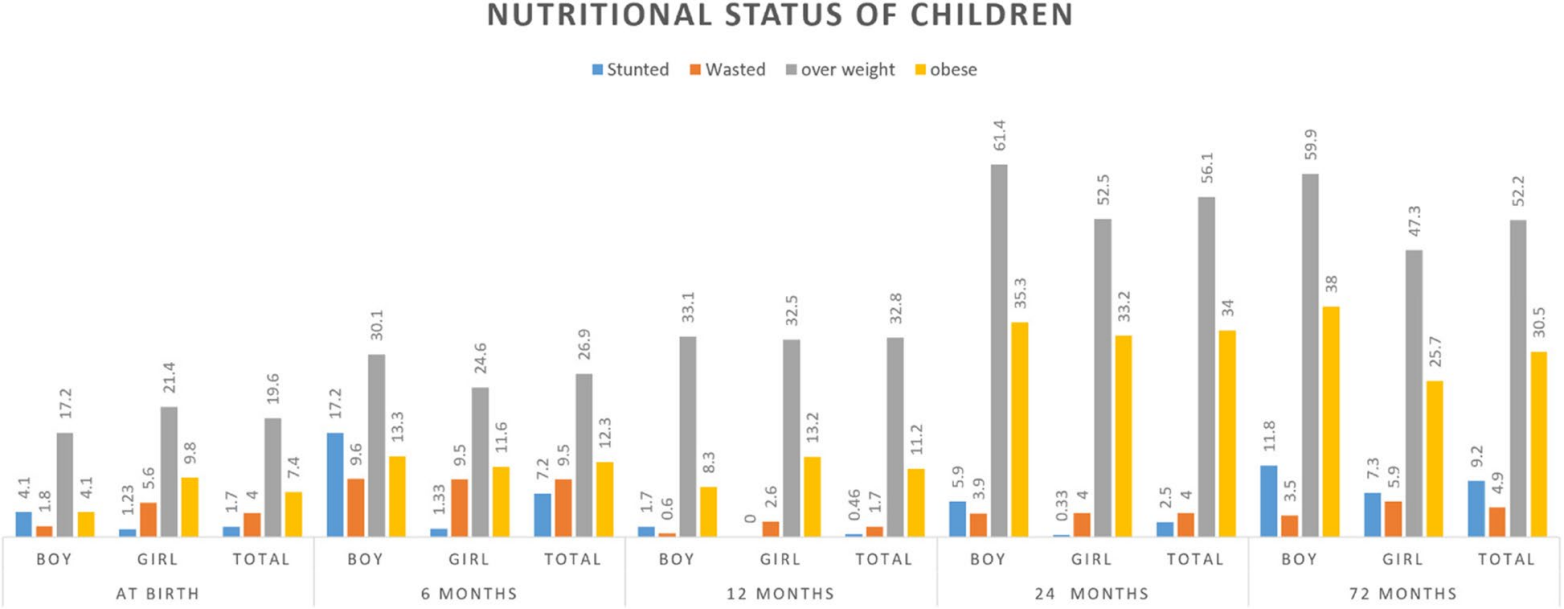
Nutritional status of total children according to age and gender in Kalar city, Kurdistan region, Iraq

The nutritional status of total children by age at five different times (birth time, months 6, 12, 24 and 6 years) has shown in Fig. 1. The highest rate of overweight was observed in boys at 24 month (61.4%). The lowest prevalence of underweight was observed in children at birth time (0.44%).

## Discussion

The present study examined the nutritional status of children under 6 years of age and related factors in Kalar city, Iraq. Our analysis indicated that the highest rate of overweight was observed in boys at 24 month (61.4%). The lowest prevalence of underweight was observed in children at the birth time (0.44%) and only mothers with academic literature had significantly higher BMI for age compared to mothers with lower education. Our findings also showed that stunting was more prevalent among boys than girls in 0–6 years old children.

Malnutrition (stunting, wasting, and overweight) in children, especially in preschool children, affect the intellectual [14, 15] and physical development [16] in childhood and increased the risk of adulthood obesity and unfavourable cardio-metabolic consequences [17], the growth trend and nutritional status of preschool children are one of the major priorities for health systems.

In a cross-sectional study [18] performed on 15,408 children under the age of 6 years in Fars province, Iran, the rates of wasting, stunting, and underweight were 8.19, 9.53, and 9.66%, respectively. Similar to our study, stunting rates were higher among boys than girls. In this study, the stunting rate was significantly associated with lower mothers' education and lower family income. Also, the large dimension of family and lack of access to health services were related to being underweight and wasting, respectively. In another study [19] of 65,908 pre-school children aged 2 to 7 years in Luoding city, China, the prevalence of overweight and obesity increased from 3.70% to 7.27% and 1.04% to 2.08%, respectively. Meanwhile, the wasting reduced from 0.91% to 0.72%, and stunting decreased from 9.29% to 5.22% from 2004 to 2013.‬‬‬ ‬This trend does not provide satisfactory results. In recent decades we have encountered a phenomenon called abdominal satiety (‬‬‬versus‬‬‬ cellular satiety), especially in low-income countries, where children appear obese while suffering from cellular malnutrition [20, 21].‬‬‬‬‬‬‬‬‬‬‬‬‬‬‬‬‬‬‬‬‬‬‬‬‬‬‬‬‬‬‬‬‬‬‬‬‬‬‬‬‬‬‬‬‬‬‬‬‬‬‬‬‬‬‬‬‬‬‬‬‬‬‬‬‬‬‬‬‬‬‬‬‬‬‬‬‬‬‬‬‬‬‬‬‬‬‬‬‬‬‬‬‬‬‬‬‬‬‬‬‬‬‬‬‬‬‬‬‬‬‬‬‬‬‬‬‬‬‬‬‬‬‬‬‬‬‬‬‬‬‬‬‬‬‬‬‬‬‬‬‬‬‬‬‬‬‬‬‬‬‬

In our study, the rate of stunting was highest among 6-month-old boys, while in the study of EN Muchina et al., [22] Which performed on 418 Kenyan children aged 0–24 months, the rate of stunting among children aged 7 to 12 months was three times higher than 0–6 months. Also, our analysis indicated that the highest rate of overweight was observed in boys at 24 month, while in this study, underweight increased with age up to 24 month and wasting was at the highest level in children 13–24 months. Also, in Chandigarh-India [23], proportions of underweight (45.5%) and stunting (81.8%) were found to be maximum among children aged 13–24 months.

In general, the difference in the prevalence of undernutrition in different parts of the world is related to various risk factors such as child’s gender, children's health care, health status and rates of infectious diseases, breastfeeding, family socioeconomic status, mother's education, pre-pregnancy weight, ethnicity, and family size.

The results indicated a higher prevalence of stunting in boys compared to girls. This finding was supported by other researchers; their studies revealed that boys due to vulnerability to childhood diseases may be at higher risk for stunting [24–26].

Our results also revealed that children of mothers with academic literature had significantly higher BMI for age compared to those of mothers without formal education. In agreement, previous studies reported a higher prevalence of undernutrition in children of mothers with lower educational levels [24, 27]. In the study of Kavosi et al. [18], the prevalence of stunting was lower in children whose mothers had an academic education than in children whose mothers had a diploma or less. Educated mothers can become more aware of their children's nutritional status and improve their children's growth process through better and more efficient health services usage and prevent malnutrition [24, 27–29]. The main strength point of our study is that it was the first study to assess the children's nutritional status in Kalar city.

The limitation of our study is that information regarding gestational age, family income, and breastfeeding status was not accurately provided.

## Conclusion

Overall, according to the standards set by WHO reference, the prevalence of stunting, and wasting among children in Kalar city ​was very low and acceptable, while the prevalence of overweight and obesity was very high. Given the serious consequences of malnutrition and the scarcity of studies determining the nutritional status of children in kurdistan region, running the studies with large sample size seems to be mandatory in this area.

## Data Availability

The data analysed in the study are available from the corresponding author on reasonable request.

## Abbreviations

HAZ: Height-for-age Z-score
BAZ: BMI-for-age Z-score
BMI: Body mass index
WHO: World health organization
SD: Standard deviation
SPSS: Statistical Package for Social Sciences
